# Surgical treatment of one traumatic carotid artery dissection: A case report and review of the literature

**DOI:** 10.1097/MD.0000000000039084

**Published:** 2024-07-26

**Authors:** Xiaojian Jia, Yuefeng Zhu

**Affiliations:** a Department of Vascular Surgery, The Fourth Affiliated Hospital Zhejiang University School of Medicine, Yiwu, Zhejiang, China; b Department of General Surgery, Sir Run Run Shaw Hospital, Zhejiang University School of Medicine, Hangzhou, Zhejiang, China.

**Keywords:** anticoagulation, antiplatelet, carotid artery dissection, cervical artery, stroke

## Abstract

**Rationale:**

Dissection of the cervical arteries is the most commonly identified cause of stroke in young patients. This report helps to investigate the etiology, diagnosis, and treatment of cervical artery dissection (CAD).

**Patient concerns:**

A 40-year-old female presented with a 3-week history of right carotid artery dissection due to a fall. The patient was admitted to the local hospital 3 weeks ago with a right neck impingement after a fall, and presented with right neck pain. The local hospital CT scan showed a dissection of the middle segment of the right common carotid artery.

**Diagnoses:**

The patient clinical manifestations and imaging tests confirmed that right carotid artery dissection.

**Interventions:**

Medical treatment with antiplatelet failed, and the CT scan showed progression of dissection. Carotid endarterectomy (CEA) was performed, and the prognosis is good.

**Outcomes:**

This patient was followed up at 1 and 6 months after the operation, CT scan showed the original stenotic vessels returned to standard diameter.

**Lessons:**

Diagnosis of CAD mainly depends on clinical manifestations and imaging. we recommend that clinicians can prescribe either anticoagulants or antiplatelet therapy. CAD can be effectively treated by surgical reconstruction, if medical treatment with anticoagulation or antiplatelet fails or if carotid aneurysms and/or high-grade carotid stenosis persisted or have newly developed.

## 1. Introduction

Cervical artery dissection (CAD) is a tear in the intima of the cervical artery that causes blood to flow into the wall of the artery to form an intramural hematoma. CAD mainly includes intercarotid artery dissection (ICAD) and vertebral artery dissection (VAD), leading to arterial stenosis, occlusion, or aneurysm.^[1]^ CAD is the most commonly identified cause of stroke in young patients.^[2]^ Herein, we report on the successful surgical treatment of a case of traumatic carotid artery dissection.

## 2. Case presentation

**Figure 1.**
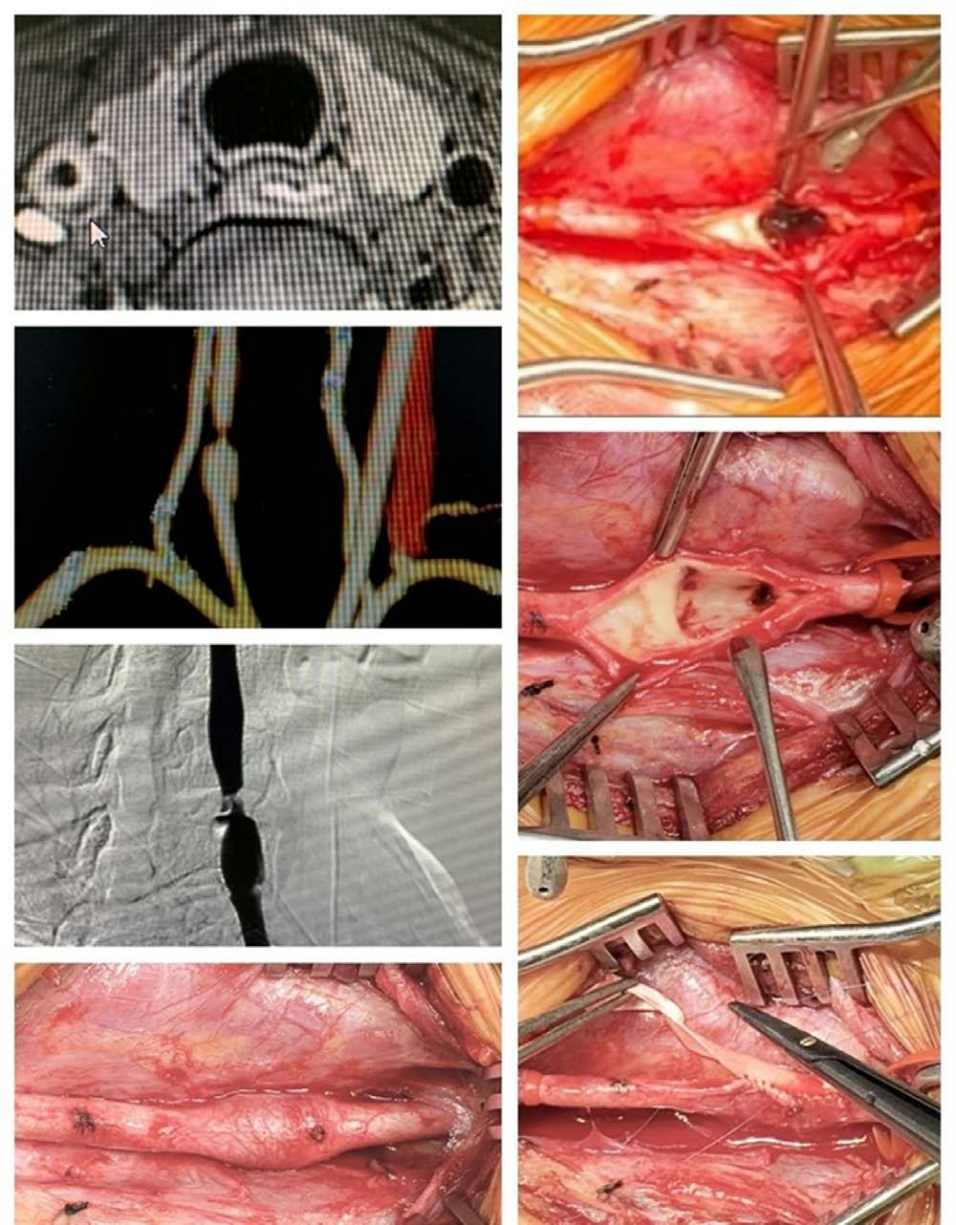
Preoperative images (CTA, MRI, and DSA) and intraoperative findings. CTA = computed tomography angiography, DSA = digital subtraction angiography, MRI = magnetic resonance imaging.

A 40-year-old female presented with a 3-week history of right carotid artery dissection due to a fall. The patient was admitted to the local hospital 3 weeks ago with a right neck impingement after a fall, and presented with right neck pain, without disturbance of consciousness or other neurological complications. The local hospital CT scan (2020.8.27) showed a dissection of the middle segment of the right common carotid artery. The patient visited the clinic in our hospital for further treatment and denied a history of hypertension, diabetes, coronary heart disease, and other medical histories. Aspirin 0.1g once a day and sulodexide 1 tablet twice a day were administered. After a month, our hospital CT scan (2020.10.8) showed the right common carotid artery dissection, and the proximal lumen of the right common carotid artery was severe stenosis. It means the dissection has progressed. The right carotid artery dissection was confirmed by preoperative examination, including magnetic resonance imaging and digital subtraction angiography. Even though there were no obvious clinical symptoms, the drug was adjusted to Aspirin 0.1g combined with rivaroxaban 10mg once a day. We recommend surgical treatment. The carotid endarterectomy (CEA) was performed, and intimal separation and thrombosis of the right carotid artery were found during the operation. Figure 1 depicts the process of endarterectomy and in situ patch augmentation (Fig. 1). The patient received dual antiplatelet therapy after surgery, recovered, without obvious discomfort, and lived a normal life. The patient was satisfied with the treatment. This patient was followed up at 1 and 6 months after the operation, CT scan showed the original stenotic vessels returned to standard diameter.

## 3. Discussion

The incidence of CAD is meager, with extracranial artery dissection (EAD) of 2.6 to 3.0 per 100,000. For intracranial artery dissection (IAD), the incidence is not available and may be lower than EAD.^[3,4]^ This paper focuses on the dissection of the extracranial cervical artery. The incidence of ICAD is about twice of that VAD.^[4]^ CAD is one of the crucial causes of IS in young and middle-aged adults. The stroke in young Fabry patients (sifap1) study in Europe involved 4208 patients aged 18 to 55 years with an event of a transient ischemic attack (TIA) or ischemic stroke (IS). 439 (10.4%) patients had CAD: 196 (50.1%) had carotid artery dissection, 195 (49.9%) had VAD, and 48 had multiple artery dissections or no information regarding the dissected artery.^[5]^

Patients with CAD usually do not have any cardiovascular risk factors, and trauma is a significant risk factor for CAD. The incidence of CAD after blunt cerebrovascular injuries (BCVI) was 1 to 2%. Major facial fractures, skull base fractures, cervical spine fractures, spinal cord injuries, or traumatic brain injuries are the anatomic injury risk factors for BCVI. Major thoracic injuries increase the incidence of carotid artery dissection, and cervical spine fractures or spinal cord injuries increase the incidence of VAD.^[6]^ Spontaneous dissection of the carotid or vertebral artery often does not have a definite history of neck trauma, but a history of a minor precipitating hyperextension or rotation of the neck when practicing yoga or other activities. In this case, the patient had a definite history of neck trauma due to carotid artery dissection caused by the impact of the right neck on an external object caused by a fall. Chiropractic manipulation of the neck has been associated with carotid artery dissection, particularly, VAD. A recent respiratory tract infection history may be another risk factor for spontaneous dissections.^[7]^ Genetic alterations have been confirmed to be associated with CAD,^[8]^ such as hyperhomocysteinemia, variation in PHACTR1 and so on.^[9,10]^ CAD is also a frequent manifestation of fibromuscular dysplasia.^[11]^ In young patients, migraine, especially migraine without aura, is consistently associated with CAD.^[12]^

CAD can lead to various local signs and symptoms, such as pain, Horner syndrome, cranial-nerve palsy^[2]^ and tinnitus.^[13]^ A single-center cohort study at the Medical University of Innsbruck shows that the incidence rate of local symptoms is about 82%.^[14]^ In this case, the right neck pain was the main manifestation, without IS. Sudden Horner syndrome, isolated headache, neck pain, or IS of the ipsilateral carotid artery may be coursed by CAD. The carotid arteries should be examined immediately.^[13]^ Subarachnoid hemorrhage (SAH) sometimes occurs in CAD, usually as a result of the dissection extending into the intracranial segment.^[2,13]^ The most common clinical manifestation in patients with CAD is cerebral ischemia (TIA or IS).^[2]^ A study with a median follow-up of 6 years showed that the incidence of TIA or IS was 73.4%.^[14]^ Also, spontaneous CAD can be presented as trigeminal neuralgia in the emergency department.^[15]^

The diagnosis of CAD is mainly based on clinical symptoms combined with imaging examination. The presence of a mural hematoma, a dissecting aneurysm, a long tapering stenosis, an intimal flap, a double lumen, or an occlusion >2 cm above the carotid bifurcation confirms the dissection of a cervical carotid or vertebral artery.^[16]^ In this case, the primary clinical manifestation of this patient was right neck pain. Combined with CT, magnetic resonance imaging, and digital subtraction angiography, the patient was diagnosed with right carotid artery dissection.

Antiplatelet therapy was first administered in this case. The clinical examination at follow-up indicated the progression of dissection. Surgical intervention, including endovascular treatment or surgical treatment, is recommended. Considering the possible thrombosis, we performed CEA to prevent the cerebral ischemic event triggered by detachment of the thrombus and intimal separation. However, thrombosis of the right carotid artery was found during the operation. The operation was successful, and the postoperative follow-up results were satisfactory. Clinical treatment strategies should be formulated according to the severity of CAD. 2021 ESO guidelines for managing extracranial and IAD recommend prescribing anticoagulants or antiplatelet therapy to treat carotid artery dissection.^[2]^ In the Cervical Artery Dissection in Stroke Study (CADISS), 250 patients were randomized to receive antiplatelet agent (AP) or anticoagulant (AC) (heparin followed by warfarin) for 3 months. Following up for twelve months, the number of recurrent strokes was low. There was no difference between treatment groups in outcome events or the recanalization rate.^[17]^ Aspirin versus anticoagulation in cervical artery dissection (TREAT-CAD) shows that Aspirin was non-inferior to vitamin K antagonists in the treatment of CAD.^[18]^ Another study of anticoagulation vs antiplatelet treatment in 370 patients with carotid and VAD shows no different rate of new or recurrent events found between 2 groups.^[19]^ In patients with residual stenosis or dissecting aneurysms, there is uncertainty over the benefits and risks of endovascular or surgical treatment.^[2]^ Surgical intervention should be recommended when the imaging follow-up shows the development of high-grade stenosis or expanding pseudoaneurysm. A single high-volume center study showed that stenting was beneficial to patients, particularly with the development of high-grade stenosis or expanding pseudoaneurysm.^[20]^ Another study showed that surgical reconstruction could prevent chronic carotid dissection from ischemic or thromboembolic complications if 6 months of anticoagulation treatment failed or progression of dissection occurred.^[21]^ In patients presenting with symptomatic CAD with acute IS within 4.5 hours of onset, alteplase should be used for intravenous thrombolysis if available and not contraindicated.^[2]^

## 4. Conclusion

The overall prognosis of CAD is good, antiplatelet and anticoagulant therapy are the basic treatment. The recurrent stroke rate at 1 year was 2% to 3% after drug therapy.^[17]^ The purpose of surgical intervention is to prevent the recurrence of IS, but the indications should be strictly controlled. In this case, the clinical manifestations of the patient were typical, the diagnosis of carotid artery dissection was confirmed. The effect of initial drug therapy failed, and the dissection progressed. In order to prevent the occurrence of stroke, surgical intervention was performed, and the curative effect was satisfactory and the prognosis was good.

## Acknowledgments

We would like to credit the patient for her participation in this case study.

## Author contributions

**Conceptualization:** Yuefeng Zhu.

**Data curation:** Xiaojian Jia.

**Writing – original draft:** Xiaojian Jia.

**Writing – review & editing:** Yuefeng Zhu.
